# Psychosocial determinants of physicians’ intention to practice euthanasia in palliative care

**DOI:** 10.1186/1472-6939-16-6

**Published:** 2015-01-22

**Authors:** Mireille Lavoie, Gaston Godin, Lydi-Anne Vézina-Im, Danielle Blondeau, Isabelle Martineau, Louis Roy

**Affiliations:** Faculty of Nursing, Laval University, Québec, G1V 0A6 Canada; Équipe de Recherche Michel-Sarrazin en Oncologie psychosociale et Soins palliatifs (ERMOS), Centre de recherche du CHU de Québec - Hôtel-Dieu de Québec, Québec, Canada; Maison Michel-Sarrazin, Quebec, Canada; CHU de Québec – Hôpital Enfant-Jésus, Quebec, Canada

**Keywords:** Euthanasia, Physician, Determinant, Intention, Palliative care

## Abstract

**Background:**

Euthanasia remains controversial in Canada and an issue of debate among physicians. Most studies have explored the opinion of health professionals regarding its legalization, but have not investigated their intentions when faced with performing euthanasia. These studies are also considered atheoretical. The purposes of the present study were to fill this gap in the literature by identifying the psychosocial determinants of physicians’ intention to practice euthanasia in palliative care and verifying whether respecting the patient’s autonomy is important for physicians.

**Methods:**

A validated anonymous questionnaire based on an extended version of the Theory of Planned Behavior was mailed to a random sample of 445 physicians from the province of Quebec, Canada.

**Results:**

The response rate was 38.3% and the mean score for intention was 3.94 ± 2.17 (range: 1 to 7). The determinants of intention among physicians were: knowing patients’ wishes (OR = 10.77; 95%CI: 1.33-86.88), perceived behavioral control—physicians’ evaluation of their ability to adopt a given behavior—(OR = 4.35; 95%CI: 1.44-13.15), moral norm—the appropriateness of adopting a given behavior according to one’s personal and moral values—(OR = 3.22; 95%CI: 1.29-8.00) and cognitive attitude—factual consequences of the adoption of a given behavior—(OR = 3.16; 95%CI: 1.20-8.35). This model correctly classified 98.8% of physicians. Specific beliefs that might discriminate physicians according to their level of intention were also identified. For instance, physicians’ moral norm was related to the ethical principle of beneficence.

**Conclusions:**

Overall, physicians have weak intentions to practice euthanasia in palliative care. Nevertheless, respecting patients’ final wishes concerning euthanasia seems to be of particular importance to them and greatly affects their motivation to perform euthanasia.

**Electronic supplementary material:**

The online version of this article (doi:10.1186/1472-6939-16-6) contains supplementary material, which is available to authorized users.

## Background

Despite the great progress made in palliative care, physicians remain confronted with the physical, psychological or spiritual limitations of their ability to alleviate suffering. In some cases it is impossible to provide suitable treatment for patients whose suffering may be seen as “unnecessary”, or even “inhuman”. Euthanasia (from the Greek *eu* [good] and *thanatos* [death]) is sometimes presented as an alternative to palliative care [1]. Yet, this practice is also radically at odds with the philosophy of palliative care [2]. Thus, it is not surprising that, although euthanasia is illegal in Canada, it remains a controversial subject among caregivers, the general population and decision makers.

Two of the major arguments in favor of euthanasia are that it puts an end to “unbearable” suffering [3] and it supports the patient’s autonomy and expressed wishes [4]. Numerous studies confirm that end-of-life patients place a high level of importance on the respect of their autonomy and wish to decide “when” and “how” they die [5–7]. Yet, a study in Flanders (Belgium) has shown that hastening death without the patient’s explicit request occurred in 1.8% of cases [8]. Moreover, euthanasia is often denounced by health professionals as an act that ends the life of another person, which is contrary to the concept of not doing harm to others (non-malfeasance, [9]) by bringing life to a definite end. Another argument against euthanasia is based on the notion of sanctity of human life, wherein life must be protected under all circumstances [10]. Finally, there is the “slippery slope” argument [9] evoking the risk of a possible “snowball effect” even if euthanasia were to be authorized only in exceptional cases.

According to previous reviews of studies on attitudes towards euthanasia among health professionals, being religious is generally associated with a negative attitude towards euthanasia [11–14]. The results for age, medical specialty and gender are more conflicting and no clear association with euthanasia is reported [11, 14]. One review identified the main reasons why some European physicians are in favor of euthanasia: 1) they believe that the patient should have the right to decide about his/her own life and death (autonomy), 2) they want to respect their patient’s desire to die with dignity, and 3) they think that the legalization of euthanasia could help avoid futile treatment [13]. On the other hand, some European physicians are against euthanasia for the following reasons: 1) they are afraid that it could result in undue pressure on vulnerable patients, 2) they are unwilling to decide about life and death and 3) they are sometimes uncertain about a patient’s prognosis [13]. Finally and not surprisingly, the authors found that physicians in Belgium and The Netherlands (two countries where euthanasia is legal) are more favorable to euthanasia compared to those from other European countries [13].

Most studies on euthanasia have explored the opinion of health professionals regarding its legalization or their experience when this act is requested. However, the studies fail to explain the intentions of caregivers faced with *performing* an act of euthanasia, which is quite different. While an individual might be in favor of legalizing euthanasia, this does not mean that he would be comfortable and willing to practice an act of euthanasia. Moreover, since most studies are atheoretical, little information is available to identify the specific beliefs underlying an intention to do so. The purposes of this study were to identify the psychosocial determinants of physicians’ intention to practice euthanasia in palliative care and to verify whether respecting the patient’s autonomy is important for physicians in order to enlighten decision makers regarding the impact of this practice on care practices and on the health professionals themselves. It is worth mentioning that in the original study, nurses’ psychosocial determinants were also identified, but only the results pertaining to physicians are reported in this article.

## Methods

### Population and sample

The population under study consisted of physicians from the province of Quebec, Canada. Physicians with a practice focusing on underage patients (e.g., pediatrics), patients with mental diseases (psychiatry), or whose job made them unlikely to care for end-of-life patients (e.g., rehabilitation, plastic surgery) were excluded.

To obtain our sample, the professional order of physicians provided a list of their active members. A random sample of 445 physicians was obtained using random digit tables. The sample was weighted according to the specialties included in the study to reflect, as closely as possible, their distribution in the province of Quebec, Canada. The study was reviewed and approved by the Ethics Committee of the Centre hospitalier universitaire (CHU) de Québec.

### Theoretical framework

**Figure 1:**
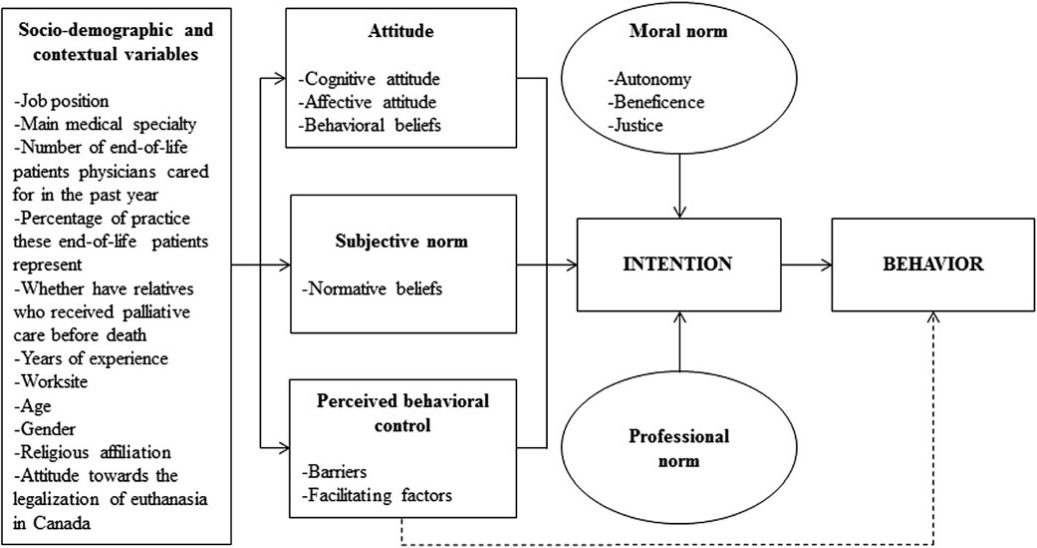
**Theoretical framework: Extended version of the Theory of Planned Behavior (Ajzen,** [15]**).** *Note*. The circles represent variables added to the Theory of Planned Behavior. The dotted line indicates that perceived behavioral control can predict behavior when the context is less volitional.

This study was guided by an extended version of the Theory of Planned Behavior (TPB, see Figure 1) [15]. Its efficacy in predicting intentions to adopt various health behaviors, including among health professionals, and the key role of intentions to predict behaviors has already been clearly established in a number of meta-analyses [16–21]. According to the TPB, behavior is predicted by intention and perceived behavioral control when the context is less volitional. Intention, in return, is formed of the following three constructs: attitude, subjective norm and perceived behavioral control (PBC). Attitude is an evaluation, either positive or negative, of the adoption of a given behavior (e.g., pleasant/unpleasant). In this study, attitudes were evaluated by using two components, cognitive and affective attitude, as suggested by Triandis [22]. Cognitive attitude refers to factual consequences (e.g., useless/useful) of the adoption of a given behavior, while affective attitude is concerned instead with emotional consequences (e.g., sad/happy). Subjective norm represents the perceived social pressure to adopt a given behavior. PBC refers to people’s evaluation of their ability to adopt a given behavior. Each construct is also related to a specific set of beliefs. Attitude is related to behavioral beliefs, subjective norm to normative beliefs and PBC to control beliefs. Behavioral beliefs refer to the perceived positive and negative consequences of the adoption of a given behavior. Normative beliefs represent how individuals believe people who are important to them would react if they adopted a given behavior (i.e., approve or disapprove). Control beliefs refer to elements that can hinder (barriers) or facilitate (facilitating factors) the adoption of a behavior. External factors such as socio-demographic variables (e.g., age, gender) can also influence the intention to adopt a given behavior through the other constructs.

New variables can be added to the TPB as long as they improve its predictive ability [15]. In this study, two variables were added, professional norm and moral norm, given that there is evidence that they are determinants of health professionals’ intention to adopt various behaviors [23, 24]. Professional norm refers to the appropriateness of adopting a behavior given one’s profession. Moral norm is a variable originating from the theory of interpersonal behavior [22]. It is related to the appropriateness of adopting a given behavior according to one’s personal and moral values. In a previous study on consent to organ donation, Blondeau et al. [25] also measured three ethical principles underlying moral norm, namely autonomy, beneficence and justice. In the present study, moral norm, as well as beneficence, justice, and autonomy were included.

In terms of external factors, the following socio-demographic and contextual variables were assessed: job position, main medical specialty, number of end-of-life patients physicians cared for in the past year and the percentage of their practice they represented, whether they had relatives who had received palliative care before their death, years of experience, worksite, age, gender, religious affiliation and attitude towards the legalization of euthanasia in Canada.

### Questionnaire development and validation

The questionnaire was developed in accordance with the methodology recommended by the author of the TPB [15]. A questionnaire based on this theory must reflect the salient beliefs of a population. To develop the belief items of the questionnaire, 21 health professionals in proximal care of dying patients (21 nurses and 8 physicians) and sharing the same socio-demographic characteristics as those of the main study sample completed a short, open-ended questionnaire containing the following questions: 1) advantages and disadvantages of practicing euthanasia in palliative care (behavioral beliefs for cognitive attitude); 2) emotions that could encourage and hinder practicing euthanasia in palliative care (behavioral beliefs for affective attitude); 3) who would approve or disapprove of this practice (normative beliefs); 4) elements that could facilitate and hinder behavioral adoption (facilitating factors and barriers); and 5) elements that could facilitate and hinder behavioral adoption given one’s professional role (professional norm).

The clinical vignettes used in the questionnaire were developed with the assistance of a nurse (IM) and a physician (LR) with many years of experience in caring for end-of-life patients. Nurses and physicians working in palliative care were also recruited to 1) ensure that the clinical vignettes adhered to clinical reality (2 nurses and 2 physicians); 2) ensure that the different clinical vignettes were suitably counterbalanced (4 nurses and 4 physicians); and 3) approve the preliminary version of the questionnaire (5 nurses and 4 physicians).

The psychometric qualities of the questionnaire were verified by means of a test-retest study. A total of 18 physicians completed the entire questionnaire twice at two-week intervals. The questionnaire had good internal consistency with all alpha coefficients above 0.70 (range: 0.83-0.96) [26]. It also had good temporal stability with all intra-class coefficients above 0.71 [27].

### Data collection

Data were collected by means of an anonymous self-administered questionnaire sent by mail (see Additional files 1 and 2 in the online version of the journal). The questionnaires were sent in mid-November 2012 with a personalized letter presenting the project, a fact sheet and with a preaddressed prepaid envelope. The return of the questionnaire was interpreted as consent to participate in the study. A first reminder was sent one week after the questionnaire was mailed and a second reminder the following week (i.e., 2 weeks after the questionnaire was mailed).

Two versions of the questionnaire were used. In both versions, prior to the items assessing the psychosocial variables, a clinical vignette with a fictional patient was presented. In the first version (A), the patient in the clinical vignette had made several explicit requests for euthanasia to the healthcare team (patient’s wishes known). In the second version (B), the patient never clearly expressed his wishes concerning the practice of an act of euthanasia (patient’s wishes unknown). This allowed us to verify whether knowledge of the patient’s position regarding euthanasia contributed to the prediction of the physicians’ intention to practice euthanasia. This also represents an indirect assessment of the inclination of physicians to respect patients’ autonomy.

Each questionnaire contained 69 items. Between 15 and 20 minutes were required to complete them. The following definition of euthanasia was provided on the cover of the questionnaire: “an act which consists in *intentionally* causing the death of a person with an incurable disease [28]”. The following definition of palliative care was also provided on the cover of the questionnaire: “an approach to care for people who are living with a life-threatening illness, regardless of their age. The focus of care is on achieving comfort and ensuring dignity for the person and maximising quality of life for the patient, family and loved ones [29]”. Participants were instructed to answer the questions by referring to the clinical vignette as if they were responsible for a case similar to the one described:

Dr Smith is Mr. Brown’s physician. The patient is 70 years old, married and a father of two. He suffers from cancer that is now generalized. Chemotherapy and radiotherapy treatments failed to stop the progression of the disease. He willingly accepted 3/3 level comfort care, which means the cessation of any curative or life-prolonging care, with the provision of palliative care.

Mr. Brown has many pulmonary, ganglionic and bone lesions that are very painful and partially responsive to analgesic treatment. He can barely hydrate and feed himself with protein shakes and his general state is very poor. He is now severely cachectic. His life expectancy is probably less than 10 days.

While discussing with the nurse, Dr Smith notices that Mr. Brown moans constantly, despite all efforts to relieve him. All possible therapeutic trials to control his pain have proven ineffective or caused intolerable side effects.

The case of Mr. Brown has already been discussed among the multidisciplinary team and with the family. Another physician confirmed the seriousness and irreversibility of his health status and the unappeasable state of his suffering. The option of sedation was also discussed, but Mr. Brown rejected this alternative. All this information is recorded in his medical file.

His condition thus raises the possibility of practising an act of euthanasia. It is important to note that this would be a *legal* act, since the practice of euthanasia in an end-of-life context would have been legalized recently in Canada.

At Mr. Brown’s bedside, Dr Smith and the nurse realize that his speech is incoherent and he can no longer assume an active role in decisions concerning his care. However, it was clearly established that Mr. Brown was apt during previous discussions concerning the possibility of cutting short his life by an act of euthanasia.

Version A (patient’s wishes known):

During those meetings, Mr. Brown made several explicit requests for euthanasia to the healthcare team.

Version B (patient’s wishes unknown):

During those meetings, Mr. Brown never clearly expressed his wishes concerning the practice of an act of euthanasia.

They were also reminded every two pages that the questions referred to a context in which the practice of euthanasia would be legally accepted. All cognitive items were measured with a 7-point Likert-type scale (strongly/somewhat/slightly disagree, neither disagree nor agree, slightly/somewhat/strongly agree), except cognitive and affective attitude which were measured with 7-point semantic differential scales (e.g., very/somewhat/slightly inappropriate, neither one, slightly/somewhat/very appropriate).

Questions pertained to each construct of the extended version of the TPB and were as follows: intention (3 items; e.g., “My intention would be to practice an act of euthanasia in a case similar to Mr. Brown’s.”), attitude (5 items for cognitive attitude; e.g., “For me, practicing an act of euthanasia in a case similar to Mr. Brown’s would be useless or useful.” and 3 items for affective attitude; e.g., “For me, practicing an act of euthanasia in a case similar to Mr. Brown’s would be uncomfortable or comfortable.”), subjective norm (3 items; e.g., “Most people important to me would accept that I practice an act of euthanasia in a case similar to Mr. Brown’s.”), perceived behavioral control (3 items; e.g., “I would be capable of practicing an act of euthanasia in a case similar to Mr. Brown’s.”), professional norm (1 item: “Practicing an act of euthanasia in a case similar to Mr. Brown’s would be compatible with my role as a physician.”), normative beliefs (4 items; e.g., “My family (spouse, father, mother, etc.) would accept that I practice an act of euthanasia in a case similar to Mr. Brown’s.”), behavioral beliefs (11 items; e.g., “Practicing an act of euthanasia in a case similar to Mr. Brown’s would cut short the patient’s pain.”), control beliefs (5 for barriers and 7 for facilitating factors; e.g., “In a case similar to Mr. Brown’s, it would be easier to practice an act of euthanasia if I believed that the patient’s condition was hopeless in terms of improvement.”), moral norm (3 items; e.g., “Practicing an act of euthanasia in a case similar to Mr. Brown’s would be acting in accordance with my principles.”), beneficence (4 items; e.g., “I would consider the following element before practicing an act of euthanasia in a case similar to Mr. Brown’s: The fact that death would represent a relief.”) and justice (4 items; e.g., “I would consider the following element before practicing an act of euthanasia in a case similar to Mr. Brown’s: The fact that this would free resources for other people in need.”).

### Statistical analyses

Determinants of intention were identified by means of multiple logistic regressions. This statistical approach was chosen, given that intention was non-normally distributed (U-shape) among the physicians. Intention was dichotomized at the median value (4.33 for physicians). In order to choose which variables to enter into the prediction model, univariate analyses were carried out on all the cognitive, socio-demographic and contextual variables [30]. Only variables with a *p* < 0.15 were entered into the regression models. Of all the variables entered, only those reaching statistical significance (*p* < 0.05) were kept in the prediction model. The variables were always entered into the regression models in the following order: step 1) direct variables of the TPB (cognitive attitude, affective attitude, subjective norm and perceived behavioral control) and knowledge of the patient’s wish concerning euthanasia, step 2) variables added to the TPB (moral norm, beneficence, justice and professional norm) and step 3) socio-demographic and contextual variables, as recommended by the author of the TPB [15]. Multiple logistic regressions were also used to identify beliefs (e.g., behavioral beliefs for attitude, normative beliefs for subjective norm or control beliefs for perceived behavioral control) discriminating participants according to their level of intention [31]. All statistical analyses were performed using SAS version 9.3 (SAS Institute, Inc., Cary, NC, USA).

## Results

### Sample characteristics

The response rate was 38.3%, comparable to similar studies among physicians [24, 32, 33] and the number of respondents represents approximately 0.8% of the total number of practicing physcians in the province of Quebec, Canada. The respondents were similar to all the physicians of the province of Quebec, Canada, in terms of gender distribution (% female: 50.5 vs. 41.5; χ^2^ = 2.84, p = 0.09), but they were younger (45.6 ± 12.8 vs. 50.6 ± 12.6; t = 4.05, *p* < 0.0001).

**Figure 2:**
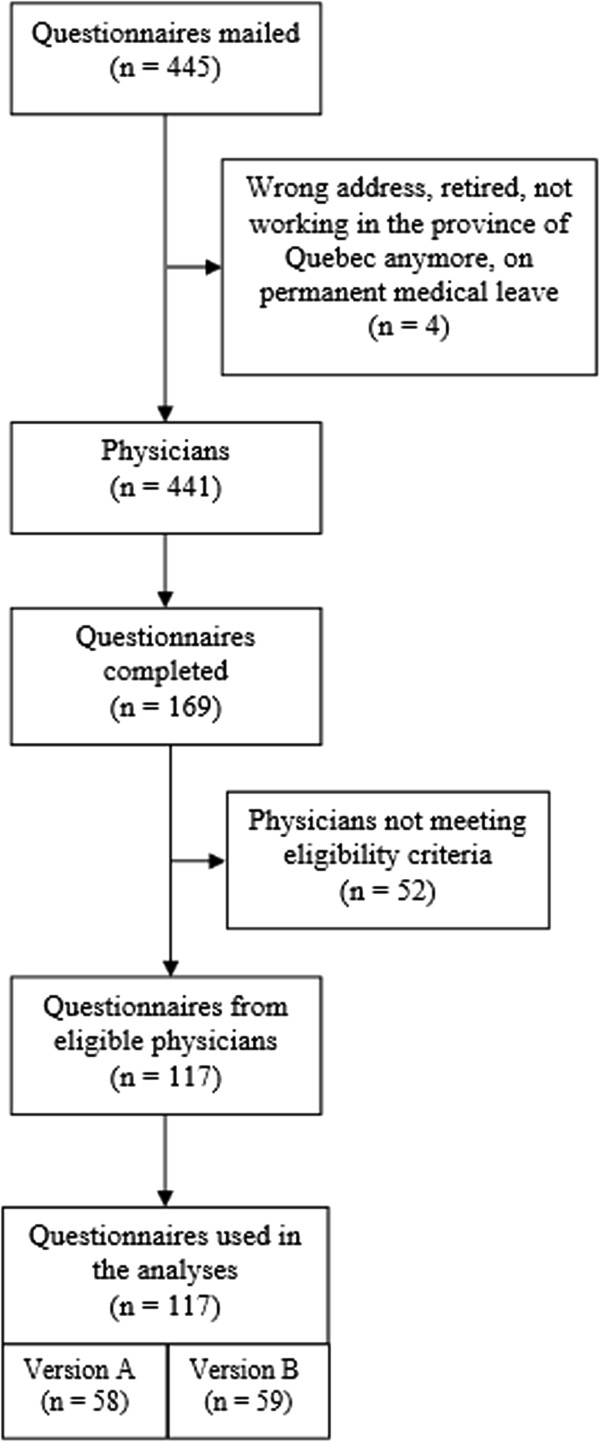
**Flow of participants.**

The main reason why physicians did not meet eligibility criteria was that they had not cared for end-of-life patients in the previous year. The flow of participants throughout the study is presented in Figure 2. More than half of the sample (57.9%) contained family physicians. The most common medical specialties were internal medicine (19.5%), oncology (12.2%), surgery (12.2%), intensive care (9.8%), cardiology (9.8%), geriatrics (7.3%) and urology (7.3%). Complete socio-demographic characteristics of the participants are presented in Table 1.

**Table 1:** **Socio-demographic characteristics of physicians (n = 117)**

Variables	Mean ± SD / %
Job position	
General practitioner or family physician	57.94%
Specialists	42.06%
Cared for end-of-life patients	
Number	27.26 ± 39.93
Percent of practice	9.97%
Relatives received palliative care before death	
Yes	47.17%
No	52.83%
Years of experience	
<1 year to 10 years	41.12%
>10 years	58.88%
Workplace	
Hospital	48.60%
Other (long-term care home, family medicine, etc.)	51.40%
Age	45.62 ± 12.79
Gender	
Male	49.53%
Female	50.47%
Religious affiliation	
Yes	64.49%
No	35.51%

### Determinants of intention to practice euthanasia in palliative care

Physicians were more or less favorably predisposed to practice euthanasia in palliative care. Sixty physicians expressed a positive intention (score > 4), 54 physicians expressed a negative intention (score < 4) and 3 physicians reported a neutral intention (score of 4). The mean score for intention was 3.94 ± 2.17 (possible scores ranging from 1 to 7). All the mean scores for the other constructs were slightly positive (>4), except for perceived behavioral control (3.67 ± 1.82), affective attitude (3.73 ± 1.69), ability to overcome barriers (3.96 ± 1.83) and justice (2.79 ± 1.56). Univariate analyses indicated that all cognitive variables were significantly related to intention (all *p*s < 0.0001). Among the socio-demographic and contextual variables, attitude towards the legalization of euthanasia in Canada (*p* < 0.0001) was significantly related to intention, whereas the percentage of practice concerned with caring for end-of-life patients (*p* = 0.0583) was near significance. All other socio-demographic and contextual variables were unrelated to intention (all *p*s > 0.15). The two versions of the questionnaire (A vs. B) were also assessed in univariate analyses to test the potential effect of knowing, or not knowing, patients’ wishes and this was significantly related to intention (*p* < 0.0001). The mean score for intention was significantly higher for the group of physicians who answered the questionnaire in which the patient’s wishes were known (version A: 4.80 ± 1.93 vs. version B: 3.09 ± 2.07, t = 4.62, *p* < 0.0001). Important differences in the percentages of physicians with a positive intention (version A: 70.69% vs. version B: 32.20%), a negative intention (version A: 25.86% vs. version B: 66.10%) and a neutral intention (version A: 3.45% vs. version B: 1.70%) were also observed between the two versions of the questionnaire. Once all variables significantly identified in the univariate analyses were entered into the multivariate analyses, the final model showed that among physicians, knowing that the patient expressed his/her wish for euthanasia (*p* = 0.0257), perceived behavioral control (*p* = 0.0092), moral norm (*p* = 0.0120) and cognitive attitude (*p* = 0.0201) were significant determinants of intention to practice euthanasia in palliative care (see Step 4 in Table 2)^a^. The model correctly classified 98.8% of physicians, which is considered excellent.

**Table 2:** **Logistic regression models for intention to practice euthanasia in palliative care among physicians (n = 117)**

	Models odds ratio (95% confidence interval)			
	Step 1	Step 2	Step 3	Step 4
Cognitive attitude	2.64 (1.03-6.74)	4.12 (1.04-16.39)	5.20 (0.86-31.37)	3.16 (1.20-8.35)
Affective attitude	2.25 (0.87-5.77)	1.38 (0.42-4.59)	1.04 (0.31-3.45)	
Subjective norm	1.36 (0.57-3.26)	0.91 (0.24-3.38)	0.86 (0.23-3.44)	
Perceived behavioral control	6.33 (2.05-19.53)	4.58 (1.03-20.43)	6.20 (1.00-38.26)	4.35 (1.44-13.15)
Moral norm		2.77 (0.71-10.78)	4.32 (0.68-27.44)	3.22 (1.29-8.00)
Beneficence		0.35 (0.07-1.80)	0.34 (0.06-2.00)	
Justice		1.50 (0.61-3.66)	1.12 (0.40-3.17)	
Professional norm		1.98 (0.75-5.20)	2.73 (0.78-9.47)	
Patient wished euthanasia	12.41 (1.56-98.99)	23.56 (1.55-356.98)	84.67 (1.46- > 999.99)	10.77 (1.33-86.88)
Percentage of end-of-life patients			1.04 (0.96-1.13)	
Index of concordance (%)	98.3	99.2	99.2	98.8

*Note*. Step 1: direct variables of the Theory of Planned behavior (TPB). Step 2: variables added to the TPB. Step 3: socio-demographic and contextual variables. Step 4: final model.

### Structure of beliefs associated with level of intention

One behavioral belief and one control belief (barrier) discriminated physicians according to their level of intention. The behavioral belief was: “Practicing an act of euthanasia in a case similar to Mr. Brown’s would honor the patient’s wishes” (OR = 2.55; 95%CI: 1.64-3.95). The control belief was: “I would feel capable of practicing an act of euthanasia in a case similar to Mr. Brown’s even if I had a sense of attachment to the patient” (OR = 2.72; 95%CI: 1.81-4.07). Physicians with a low level of intention had significantly lower scores on these two beliefs compared to those with a high level of intention (behavioral belief: 3.32 ± 1.97 vs. 6.19 ± 1.22, t = −9.48, *p* < 0.0001; control belief: 2.91 ± 1.93 vs. 6.08 ± 0.95, t = −11.29, *p* < 0.0001).

The physicians’ belief about honoring the patient’s wishes was very much influenced by the version of questionnaire they answered. Among the physicians who received the questionnaire in which the patient’s wishes were known (version A), 89.66% agreed with this statement compared to only 30.51% among those who answered the questionnaire in which the patient’s wishes were not known (version B). In fact, when the patient’s wishes were not known, the majority of physicians disagreed with the item (52.54%) or adopted a neutral position (16.95%).

Given that moral norm is a determinant of intention, its underlying moral principles (beneficence and justice) were also investigated. One item of beneficence discriminated physicians according to their level of intention. The belief was: “I would consider the following element before practicing an act of euthanasia in a case similar to Mr. Brown’s: the fact that this would be acting in the best interest of an end-of-life person” (OR = 2.95; 95% CI: 1.95-4.47). Physicians with a low intention had significantly lower scores on this item compared to those with a high level of intention (3.75 ± 2.10 vs. 6.37 ± 0.85, t = −8.86, *p* < 0.0001). No justice items significantly discriminated physicians according to their level of intention (all *p*s > 0.05).

## Discussion

Most studies on euthanasia have explored the opinion of health professionals regarding its legalization and whether they support this act. Few studies have assessed physicians’ actual intention to practice euthanasia. In the present study, the results showed that physicians had, overall, a low intention to practice euthanasia in palliative care if this practice were legal, with a mean score slightly on the negative side (3.94 ± 2.17). In fact, the sample was nearly divided in half concerning who expressed a positive or a negative intention, indicating polarized positions on the topic. This result is similar to a previous review of euthanasia which reported that less than 50% of US physicians support euthanasia or physician-assisted suicide [11].

The results also confirmed the efficacy of using an extended version of the TPB to explain the intention to adopt this behavior. The prediction model included not only variables from the TPB, but also variables added to the model. Moreover, the results provided further evidence that moral norm is defined by the ethical principle of beneficence. Finally, respecting patients’ autonomy—in this case, their final wishes concerning euthanasia—seemed of uttermost importance to physicians. The following paragraphs will discuss physicians’ intention in light of each of these variables.

Perceived behavioral control (PBC) was one of the most important determinants of physicians’ intention to practice euthanasia, which supports the premises of the TPB [15, 34, 35]. This result indicates that physicians would be more inclined to practice euthanasia if they thought that they had the ability to perform this act. This could also be interpreted as a sign that physicians want to be sure they have the medical skills needed to perform an act of euthanasia before considering performing it. It should be noted that in previous studies based on the TPB, PBC was also an important determinant of intention among physicians to perform a range of different behaviors [24, 33, 36, 37].

Another determinant of physicians’ intention to practice euthanasia was attitude. Interestingly, only cognitive attitude contributed to physicians’ motivation to perform an act of euthanasia, not its emotional equivalent, affective attitude. This suggests that in the case of euthanasia, when physicians form their intention, they rely mainly on their rational side and on the factual—rather than the emotional—consequences that practicing this act would have on them. Some authors explain this phenomenon as a struggle between desire and reason [38]. In fact, in a study among Dutch physicians who have practiced euthanasia, a physician mentioned “There’s a heart and mind conflict” to describe how he felt between the time a patient first asked him for euthanasia and the time the patient actually received it [39]. Moreover, a previous study among physicians also identified only the cognitive component of attitude as a determinant of intention to adopt a medically-related behavior (i.e., shared decision-making in home care programs) [40].

One variable added to the TPB that significantly contributed to the prediction of physicians’ intention was moral norm. While this variable has already been associated with physicians’ motivation in previous studies [21, 24], it is the first time that it has been linked to performing euthanasia. More precisely, physicians’ moral norm was related to the ethical principle of beneficence. This result replicates those of a previous study on consent to organ donation among the general population [25]. In this latter study, three ethical principles (autonomy, beneficence and justice) were tested as potential principles underlying moral norm; only beneficence was significantly related to this construct. This suggests that physicians would be motivated to practice euthanasia if they perceived that this act were in agreement with their personal values and principles and if they believed that this would also be in the best interests of an end-of-life person.

Another ethical principle that had a significant impact on physicians’ intention to practice euthanasia was patients’ autonomy. This was assessed by using the two versions of the questionnaire. When physicians answered the questionnaire in which the patient had requested euthanasia (version A), their motivation to perform this act was much greater compared to those who completed the questionnaire in which the patient’s wishes were not known (version B). In fact, knowing or not knowing the patient’s wishes was the main determinant of physicians’ intention to practice euthanasia. In addition, the behavioral belief underlying their cognitive attitude was that performing euthanasia would honor the patient’s wishes. Again, the score on this item was related to the version of questionnaire received. More physicians who received the questionnaire in which the patient’s wishes were known agreed with this item compared to the physicians who received the other version. Overall, these results indicate that the main reason why physicians would be motivated to practice euthanasia would be to respect patients’ autonomy, by fulfilling their wish to die by a lethal injection. Similarly, in a previous review of European physicians’ attitudes towards euthanasia and physician-assisted suicide, the right of the patient to decide about his/her own life and death was one reason why they mentioned being in favor of euthanasia [13]. Still, it is important to note that almost one third of the physicians (32.2%) in our sample intended to practice euthanasia in the absence of the patient’s wishes. This clearly emphasizes the importance of carefully controlling this practice, and to some extent, the validity of the slippery slope argument.

Unlike previous reviews on physicians’ attitudes toward euthanasia, no socio-demographic and contextual variables contributed to explain physicians’ intention in this study. In particular, religious affiliation did not contribute to physicians’ intention whereas in the past, it had systematically been associated with a negative attitude towards euthanasia [11–14]. Contrary to a review among US physicians [11], medical specialty was not a determinant of intention, although another review among European physicians reported conflicting results for this variable [13]. This latter review also reported conflicting results for age and gender [13]. Finally, in the present study, physicians’ attitude toward the legalization of euthanasia in Canada was another variable unrelated to intention in the multivariate analyses. While at first these results may appear to run counter to common sense, they support the assumptions of the TPB. According to this theory, socio-demographic variables are external factors whose influence on intention should be filtered (or mediated) through psychosocial constructs, such as subjective norm, attitude and PBC; they should not be direct determinants of intention. Accordingly, these results provide additional support for the use of the TPB to identify physicians’ intention to practice euthanasia in palliative care.

One last result is worth mentioning concerning the percentage of physicians favorably predisposed to practice euthanasia. This study indicates that physicians are somewhat divided in terms of their motivation to perform this act: approximately half reported a positive intention and the other half, a negative intention. In the case of physicians who are part of the same medical team, hospital or hospice, could these polarized positions on euthanasia lead to disagreements or strained work relations? Could physicians who are in favor of euthanasia be perceived as “cruel” for having the intention to end the life of a patient? Conversely, could physicians who are against euthanasia be perceived as “cruel” for *not* having the intention to end the life of a patient who is suffering? Could some physicians feel pressured to perform euthanasia? Already, in countries where euthanasia is legal, physicians have reported significant feelings of discomfort, isolation and self-imposed sacrifice [41, 42]. These questions would definitely be interesting to investigate in future studies, and physicians who report such negative consequences following euthanasia should receive the help they need.

In sum, the results of this study further add to the increasing evidence of the efficacy of using an extended version of the TPB to study health professionals’ intentions and behaviors. In 2008, a systematic review had already identified the TPB as a promising theory to predict their behaviors [21]. The novelty here is the application of this theory to study physicians’ intention to practice euthanasia. In fact, most research in the field of palliative care is atheoretical. Yet increasingly more authors are calling for more theory-based studies, given that the use of theories presents many advantages [43–46]. For instance, theory can guide the selection of determinants to be tested as potential predictors of intention or behavior [47]. The use of theory can also facilitate the replication of previous findings crucial to increase scientific knowledge about certain behaviors [48]. Additionally, this can provide a basis for refining and developing better theories [47].

Strengths of this study include the use a psychosocial theory to study physicians’ intention to practice euthanasia in palliative care and the rigorous methodology used to develop and validate the survey instrument. One limitation of the study is the low response rate. Yet, according to some authors, low response rates are more acceptable when the topic is controversial, such as in the case of euthanasia [49, 50]. Respondents were also compared to the population under study to ascertain whether they were similar in terms of gender distribution and age. Although the respondents were younger compared to physicians from the province of Quebec, Canada, the results were similar when controlling for age in the analyses. Still, the possibility of a response bias cannot be completely ruled out.

## Conclusions

This study has the distinction of identifying the ethical principles underlying physicians’ moral norm and intention. It is hoped that this will encourage more theoretical research on physicians’ motivation to practice euthanasia and on the ethical principles related to this practice. Finally, to gain more insight, future studies conducted in countries where euthanasia is legal could measure the behavior itself to verify whether the determinants of intention are similar to those of behavior.

## Endnote

^a^Given that our respondents were younger than the population of physicians of the province of Quebec, Canada, we verified whether the results were similar when controlling for age; no change was observed in the predictive model (data not shown). In fact, age was barely correlated to intention (r = −0.01).

## Electronic supplementary material

Additional file 1: **Physicians’ questionnaire with the patient’s wishes known (version A).** (DOC 301 KB)

Additional file 2: **Physicians’ questionnaire with the patient’s wishes not known (version B).** (DOC 301 KB)
