# Supplementary material for: Psychosocial determinants of physicians’ intention to practice euthanasia in palliative care
Source: BMC Med Ethics. 2015 Jan 22;16:6. doi: 10.1186/1472-6939-16-6 (PMC4417253; doi:10.1186/1472-6939-16-6)
Supplement: Supplementary file 1 — Additional file 1: Physicians’ questionnaire with the patient’s wishes known (version A). (DOC 301 KB) [file 12910_2014_333_MOESM1_ESM.doc]

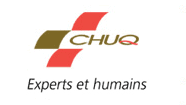

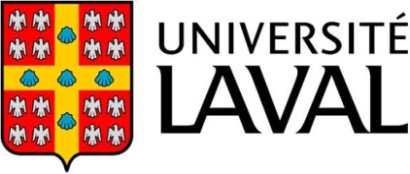


**study of the determinants of nurses’ and physicians’ intention to practiSe or not an act of euthanasia in a context of palliative care**

***Questionnaire***

**Definitions**

Euthanasia refers to an act which consists in *intentionally* causing the death of a person with an incurable disease.

Palliative care is an approach to care for people who are living with a life-threatening illness, regardless of their age. The focus of care is on achieving comfort and ensuring dignity for the person and maximizing quality of life for the patient, family and loved ones.

**Date: _______________**

| **A** |  |  |
| --- | --- | --- |

| **INSTRUCTIONS TO COMPLETE THE QUESTIONNAIRE** |
| --- |

1. You may feel that some questions are repetitive. However, it is very important to answer all of them.
2. To answer the questions, enter your answer in the place best reflecting your opinion or situation.

**Example**

It snows a lot in Quebec during the winter.

|  |  |  |  |  |  |  |  |  |  |  | **✓** |  |  |
| --- | --- | --- | --- | --- | --- | --- | --- | --- | --- | --- | --- | --- | --- |
|  | Strongly disagree |  | Somewhat disagree |  | Slightly disagree |  | Neither disagree nor agree |  | Slightly  agree |  | Somewhat agree |  | Strongly agree |

1. Take your time to read each question or statement carefully and **provide the answer best reflecting what you currently think.**
2. There are no right or wrong answers.
3. Your answers will remain confidential.

**Sociodemographic data**

1. Do you hold a license to practise from the Collège des médecins du Québec (CMQ)?

Yes

No **→** The questionnaire ends here. Please return it to us in the envelope provided.

1. What position do you currently hold?

General practitioner / family physician

Specialist physician

Specify your main specialty: _____________________________

1. Are the majority of your patients adults?

Yes

No **→** The questionnaire ends here. Please return it to us in the envelope provided.

1. Do you work mainly in one of the following domains of practice (or unit)?

| - Adolescent medicine - Anatomical pathology - Anesthesiology - Clinical immunology and allergy - Community medicine - Dermatology - Diagnostic radiology - Medical biochemistry - Endocrinology and metabolism | - Forensic pathology - General pathology - Hematological pathology - Maternal-fetal medicine - Medical genetics - Medical microbiology and infectious diseases - Neonatal-perinatal medicine - Neuropathology | - Nuclear medicine - Occupational medicine - Ophthalmology - Orthopaedics - Pediatrics - Physical medicine and rehabilitation - Plastic surgery - Psychiatry - Rheumatology |
| --- | --- | --- |

Yes **→** The questionnaire ends here. Please return it to us in the envelope provided.

No

1. In the past year, approximately how many end-of-life patients did you care for? ______________

**→** If you answered "none" at Item 5, the questionnaire ends here. Please return it to us in the envelope provided.

a. Approximately what percentage of your practice do these patients represent? _______________%

**Sociodemographic data (continued)**

1. Did any of your relatives receive palliative care before their death?

Yes

No

1. How many years of experience do you have as a physician?

Less than one year

Between 1 and 5 years

Between 6 and 10 years

Between 11 and 15 years

Between 16 and 25 years

More than 26 years

1. Where do you currently work?

*[Check all the boxes that apply]*

Hospital complex or hospital

CLSC

Long-term care home (CHSLD)

Centre de santé et de services sociaux (CSSS)

Family medicine group (GMF)

Family medicine unit (UMF)

Other: _____________________________

1. What is your age: ________
2. What is your gender?

Male

Female

1. Do you have any religious affiliation?

Yes. Specify which: _____________________________

No

**Clinical vignette**

Dr. Smith is Mr. Brown’s physician. The patient is 70 years old, married and a father of two. Mr. Brown suffers from cancer that is now generalized. Chemotherapy and radiotherapy treatments failed to stop the progression of the disease. He willingly accepted 3/3 level comfort care, which means the cessation of any curative or life-prolonging care, with the provision of palliative care.

Mr. Brown has many pulmonary, ganglionic and bone lesions that are very painful and partially responsive to analgesic treatment. He can barely hydrate and feed himself with protein shakes and his general state is very poor. He is now severely cachectic. His life expectancy is probably less than 10 days.

While discussing with the nurse, Dr. Smith notices that Mr. Brown moans constantly, despite all efforts to relieve him. All possible therapeutic trials to control his pain have proven ineffective or caused intolerable side effects.

The case of Mr. Brown has already been discussed among the multidisciplinary team and with the family. Another physician confirmed the seriousness and irreversibility of his health status and the unappeasable state of his suffering. The option of sedation was also discussed, but Mr. Brown rejected this alternative. All this information is recorded in his medical file.

His condition thus raises the possibility of practising an act of euthanasia. It is important to note that this would be a *legal* act, since the practice of euthanasia in an end-of-life context would have been legalized recently in Canada.

At Mr. Brown’s bedside, Dr. Smith and the nurse realize that his speech is incoherent and he can no longer assume an active role in the decisions concerning his care. However, it was clearly established that Mr. Brown was apt during previous discussions concerning the possibility of cutting short his life by an act of euthanasia.

During those meetings, Mr. Brown made several explicit requests for euthanasia to the healthcare team.

| **Please answer the following questions by referring to the clinical vignette**  **as if you were responsible for a case similar to Mr. Brown’s.**  **May we remind you that these questions refer to a context in which**  **the practice of euthanasia would be legally accepted.** |
| --- |

1. My intention would be to practise an act of euthanasia in a case similar to Mr. Brown’s.

|  |  |  |  |  |  |  |  |  |  |  |  |  |  |
| --- | --- | --- | --- | --- | --- | --- | --- | --- | --- | --- | --- | --- | --- |
|  | Strongly disagree |  | Somewhat disagree |  | Slightly disagree |  | Neither disagree nor agree |  | Slightly agree |  | Somewhat agree |  | Strongly agree |

1. Most people important to me would accept that I practise an act of euthanasia in a case similar to Mr. Brown’s.

|  |  |  |  |  |  |  |  |  |  |  |  |  |  |
| --- | --- | --- | --- | --- | --- | --- | --- | --- | --- | --- | --- | --- | --- |
|  | Strongly disagree |  | Somewhat disagree |  | Slightly disagree |  | Neither disagree nor agree |  | Slightly agree |  | Somewhat agree |  | Strongly agree |

1. For me, practising an act of euthanasia in a case similar to Mr. Brown’s would be…

|  |  |  |  |  |  |  |  |  |  |  |  |  |  |
| --- | --- | --- | --- | --- | --- | --- | --- | --- | --- | --- | --- | --- | --- |
|  | Very difficult |  | Somewhat difficult |  | Slightly difficult |  | Neither difficult nor easy |  | Slightly easy |  | Somewhat easy |  | Very  easy |

1. Practising an act of euthanasia in a case similar to Mr. Brown’s would be acting in accordance with my principles.

|  |  |  |  |  |  |  |  |  |  |  |  |  |  |
| --- | --- | --- | --- | --- | --- | --- | --- | --- | --- | --- | --- | --- | --- |
|  | Strongly disagree |  | Somewhat disagree |  | Slightly disagree |  | Neither disagree nor agree |  | Slightly agree |  | Somewhat agree |  | Strongly agree |

1. My family (spouse, father, mother, etc.) would accept that I practise an act of euthanasia in a case similar to Mr. Brown’s.

|  |  |  |  |  |  |  |  |  |  |  |  |  |  |
| --- | --- | --- | --- | --- | --- | --- | --- | --- | --- | --- | --- | --- | --- |
|  | Strongly disagree |  | Somewhat disagree |  | Slightly disagree |  | Neither disagree nor agree |  | Slightly agree |  | Somewhat agree |  | Strongly agree |

1. For me, practising an act of euthanasia in a case similar to Mr. Brown’s would be …

*[Check the appropriate box for* ***each*** *of the following eight statements]*

|  | Very | Somewhat | Slightly | Neither one | Slightly | Somewhat | Very |  |
| --- | --- | --- | --- | --- | --- | --- | --- | --- |
| **Useless** |  |  |  |  |  |  |  | **Useful** |
| **Harmful** |  |  |  |  |  |  |  | **Beneficial** |
| **Unsafe** |  |  |  |  |  |  |  | **Safe** |
| **Inappropriate** |  |  |  |  |  |  |  | **Appropriate** |
| **Irrational** |  |  |  |  |  |  |  | **Rational** |
| **Guilt-ridden** |  |  |  |  |  |  |  | **Guilt-free** |
| **Uncomfortable** |  |  |  |  |  |  |  | **Comfortable** |
| **Unsatisfying** |  |  |  |  |  |  |  | **Satisfying** |

1. It would be up to me to practise an act of euthanasia in a case similar to Mr. Brown’s.

|  |  |  |  |  |  |  |  |  |  |  |  |  |  |
| --- | --- | --- | --- | --- | --- | --- | --- | --- | --- | --- | --- | --- | --- |
|  | Strongly disagree |  | Somewhat disagree |  | Slightly disagree |  | Neither disagree nor agree |  | Slightly agree |  | Somewhat agree |  | Strongly agree |

1. The physicians with whom I work would accept that I practise an act of euthanasia in a case similar to Mr. Brown’s.

|  |  |  |  |  |  |  |  |  |  |  |  |  |  |
| --- | --- | --- | --- | --- | --- | --- | --- | --- | --- | --- | --- | --- | --- |
|  | Strongly disagree |  | Somewhat disagree |  | Slightly disagree |  | Neither disagree nor agree |  | Slightly agree |  | Somewhat agree |  | Strongly agree |

1. The chances that I practise an act of euthanasia in a case similar to Mr. Brown’s would be…

|  |  |  |  |  |  |  |  |  |  |  |  |  |  |
| --- | --- | --- | --- | --- | --- | --- | --- | --- | --- | --- | --- | --- | --- |
|  | Very  low |  | Somewhat low |  | Slightly  low |  | Neither low  nor high |  | Slightly  high |  | Somewhat high |  | Very  high |

**Reminder**

**These questions are in line with a context whereby the practice of euthanasia**

**would be legally accepted.**

1. If I practised an act of euthanasia in a case similar to Mr. Brown’s, most people who are important to me would…

|  |  |  |  |  |  |  |  |  |  |  |  |  |  |
| --- | --- | --- | --- | --- | --- | --- | --- | --- | --- | --- | --- | --- | --- |
|  | Strongly disagree |  | Somewhat disagree |  | Slightly disagree |  | Neither disagree nor agree |  | Slightly agree |  | Somewhat agree |  | Strongly agree |

1. In a case similar to Mr. Brown’s, I would practise an act of euthanasia.

|  |  |  |  |  |  |  |  |  |  |  |  |  |  |
| --- | --- | --- | --- | --- | --- | --- | --- | --- | --- | --- | --- | --- | --- |
|  | Strongly disagree |  | Somewhat disagree |  | Slightly disagree |  | Neither disagree nor agree |  | Slightly agree |  | Somewhat agree |  | Strongly agree |

1. My personal values would encourage me to practise an act of euthanasia in a case similar to Mr. Brown’s.

|  |  |  |  |  |  |  |  |  |  |  |  |  |  |
| --- | --- | --- | --- | --- | --- | --- | --- | --- | --- | --- | --- | --- | --- |
|  | Strongly disagree |  | Somewhat disagree |  | Slightly disagree |  | Neither disagree nor agree |  | Slightly agree |  | Somewhat agree |  | Strongly agree |

1. I would feel **capable** of practising an act of euthanasia in a case similar to Mr. Brown’s **even if**…

|  | Strongly disagree | Somewhat disagree | Slightly disagree | Neither one | Slightly agree | Somewhat agree | Strongly agree |
| --- | --- | --- | --- | --- | --- | --- | --- |
| a) I had a sense of attachment to the patient. |  |  |  |  |  |  |  |
| b) The patient were unconscious. |  |  |  |  |  |  |  |
| c) I felt like I was deliberately causing the death of the patient. |  |  |  |  |  |  |  |
| d) The family disagreed with the patient’s decision. |  |  |  |  |  |  |  |
| e) This is an irrevocable decision. |  |  |  |  |  |  |  |

1. My co-workers would accept that I practise an act of euthanasia in a case similar to Mr. Brown’s.

|  |  |  |  |  |  |  |  |  |  |  |  |  |  |
| --- | --- | --- | --- | --- | --- | --- | --- | --- | --- | --- | --- | --- | --- |
|  | Strongly disagree |  | Somewhat disagree |  | Slightly disagree |  | Neither disagree nor agree |  | Slightly agree |  | Somewhat agree |  | Strongly agree |

1. I would be capable of practising an act of euthanasia in a case similar to Mr. Brown’s.

|  |  |  |  |  |  |  |  |  |  |  |  |  |  |
| --- | --- | --- | --- | --- | --- | --- | --- | --- | --- | --- | --- | --- | --- |
|  | Strongly disagree |  | Somewhat disagree |  | Slightly disagree |  | Neither disagree nor agree |  | Slightly agree |  | Somewhat agree |  | Strongly agree |

1. Practising an act of euthanasia in a case similar to Mr. Brown’s…

|  | Strongly disagree | Somewhat disagree | Slightly disagree | Neither one | Slightly agree | Somewhat agree | Strongly agree |
| --- | --- | --- | --- | --- | --- | --- | --- |
| a) Would cut short the patient’s pain. |  |  |  |  |  |  |  |
| b) Would cut short the pain of the patient’s family. |  |  |  |  |  |  |  |
| c) Would honour the patient’s wishes. |  |  |  |  |  |  |  |
| d) Could cause conflict among family members. |  |  |  |  |  |  |  |
| e) Could cause conflict among the medical team and the family. |  |  |  |  |  |  |  |
| f) Could cause conflict among members of the medical team. |  |  |  |  |  |  |  |
| g) Would appease the medical team’s feelings of helplessness. |  |  |  |  |  |  |  |
| h) Would appease the family’s feelings of helplessness. |  |  |  |  |  |  |  |
| i) Would be an expression of my empathy. |  |  |  |  |  |  |  |
| j) Would be an act of compassion. |  |  |  |  |  |  |  |
| k) Would make me sad. |  |  |  |  |  |  |  |

**Reminder**

**These questions are in line with a context whereby the practice of euthanasia**

**would be legally accepted.**

1. Practising an act of euthanasia in a case similar to Mr. Brown’s would be compatible with my role as a physician.

|  |  |  |  |  |  |  |  |  |  |  |  |  |  |
| --- | --- | --- | --- | --- | --- | --- | --- | --- | --- | --- | --- | --- | --- |
|  | Strongly disagree |  | Somewhat disagree |  | Slightly disagree |  | Neither disagree nor agree |  | Slightly agree |  | Somewhat agree |  | Strongly agree |

1. People of great importance to me think that I should practise an act of euthanasia in a case similar to Mr. Brown’s.

|  |  |  |  |  |  |  |  |  |  |  |  |  |  |
| --- | --- | --- | --- | --- | --- | --- | --- | --- | --- | --- | --- | --- | --- |
|  | Strongly disagree |  | Somewhat disagree |  | Slightly disagree |  | Neither disagree nor agree |  | Slightly agree |  | Somewhat agree |  | Strongly agree |

1. In a case similar to Mr. Brown’s, it would be **easier** to practise an act of euthanasia if…

|  | Strongly disagree | Somewhat disagree | Slightly disagree | Neither one | Slightly agree | Somewhat agree | Strongly agree |
| --- | --- | --- | --- | --- | --- | --- | --- |
| a) The patient were unconscious. |  |  |  |  |  |  |  |
| b) I had the consent of the patient’s family. |  |  |  |  |  |  |  |
| c) I believed that the patient did not have any quality of life left. |  |  |  |  |  |  |  |
| d) I believed that the patient did not have any dignity left. |  |  |  |  |  |  |  |
| e) I believed that the patient’s condition was hopeless in terms of improvement. |  |  |  |  |  |  |  |
| f) The act of euthanasia was accepted by society. |  |  |  |  |  |  |  |
| g) The responsibility of the decision was shared. |  |  |  |  |  |  |  |

1. Practising an act of euthanasia in a case similar to Mr. Brown’s would be compatible with my moral values.

|  |  |  |  |  |  |  |  |  |  |  |  |  |  |
| --- | --- | --- | --- | --- | --- | --- | --- | --- | --- | --- | --- | --- | --- |
|  | Strongly disagree |  | Somewhat disagree |  | Slightly disagree |  | Neither disagree nor agree |  | Slightly agree |  | Somewhat agree |  | Strongly agree |

1. The nurses with whom I work would accept that I practise an act of euthanasia in a case similar to Mr. Brown’s.

|  |  |  |  |  |  |  |  |  |  |  |  |  |  |
| --- | --- | --- | --- | --- | --- | --- | --- | --- | --- | --- | --- | --- | --- |
|  | Strongly disagree |  | Somewhat disagree |  | Slightly disagree |  | Neither disagree nor agree |  | Slightly agree |  | Somewhat agree |  | Strongly agree |

1. I would consider the following elements before practising an act of euthanasia in a case similar to Mr. Brown’s…

|  | Strongly disagree | Somewhat disagree | Slightly disagree | Neither one | Slightly agree | Somewhat agree | Strongly agree |
| --- | --- | --- | --- | --- | --- | --- | --- |
| a) The fact that death would represent a relief. |  |  |  |  |  |  |  |
| b) The fact that this would help someone. |  |  |  |  |  |  |  |
| c) The fact that this would preserve the dignity of the patient. |  |  |  |  |  |  |  |
| d) The fact that this would be acting in the best interests of an end-of-life person. |  |  |  |  |  |  |  |
| e) The fact that this would mitigate the feelings of unfairness that sick people can feel. |  |  |  |  |  |  |  |
| f) The fact that this would lower healthcare costs. |  |  |  |  |  |  |  |
| g) The fact that this would reduce the burden on society. |  |  |  |  |  |  |  |
| h) The fact that this would free resources for other people in need. |  |  |  |  |  |  |  |

One last question:

1. What is your attitude towards the **legalization** of euthanasia in Canada?

|  |  |  |  |  |  |  |  |  |  |  |  |  |  |
| --- | --- | --- | --- | --- | --- | --- | --- | --- | --- | --- | --- | --- | --- |
|  | Strongly unfavourable |  | Somewhat unfavourable |  | Slightly unfavourable |  | Neither unfavourable nor favourable |  | Slightly favourable |  | Somewhat favourable |  | Strongly favourable |

**Thank you for your cooperation!**

**Comments:**

|  |
| --- |
|  |
|  |
|  |
|  |
|  |
|  |
|  |
|  |
|  |
|  |
|  |
|  |
|  |
|  |
|  |
